# Systematic evaluation of imaging techniques and baseline characteristics in patients with suspected vasculitis

**DOI:** 10.1016/j.ejro.2022.100445

**Published:** 2022-10-12

**Authors:** Vitali Koch, Julia Abt, Leon D. Gruenewald, Katrin Eichler, Tommaso D’Angelo, Simon S. Martin, Moritz H. Albrecht, Axel Thalhammer, Christian Booz, Ibrahim Yel, Simon Bernatz, Scherwin Mahmoudi, Marc Harth, Wojciech Derwich, Thomas J. Vogl, Daphne Gray, Tatjana Gruber-Rouh, Georg Jung

**Affiliations:** a Department of Diagnostic and Interventional Radiology, University Hospital Frankfurt, Frankfurt am Main, Germany; b Department of Biomedical Sciences and Morphological and Functional Imaging, University Hospital Messina, Messina, Italy; c Department of Vascular Surgery, University Hospital Frankfurt, Frankfurt am Main, Germany

**Keywords:** Vasculitis, Diagnostic imaging, Magnetic resonance imaging, Positron emission tomography

## Abstract

**Purpose:**

To assess the diagnostic value of different imaging modalities in distinguishing systemic vasculitis from other internal and immunological diseases.

**Methods:**

This retrospective study included 134 patients with suspected vasculitis who underwent ultrasound, magnetic resonance imaging (MRI), or ^18^F-fluorodeoxyglucose positron emission tomography/computed tomography (^18^F-FDG PET/CT) between 01/2010 and 01/2019, finally consisting of 70 individuals with vasculitis. The main study parameter was the confirmation of the diagnosis using one of the three different imaging modalities, with the adjudicated clinical and histopathological diagnosis as the gold standard. A secondary parameter was the morphological appearance of the vessel affected by vasculitis.

**Results:**

Patients with systemic vasculitis had myriad clinical manifestations with joint pain as the most common symptom. We found significant correlations between different imaging findings suggestive of vasculitis and the final adjudicated clinical diagnosis. In this context, on MRI, vessel wall thickening, edema, and diameter differed significantly between vasculitis and non-vasculitis groups (*p* < 0.05). Ultrasound revealed different findings that may serve as red flags in identifying patients with vasculitis, such as vascular occlusion or halo sign (*p* = 0.02 vs. non-vasculitis group). Interestingly, comparing maximal standardized uptake values from PET/CT examinations with vessel wall thickening or vessel diameter did not result in significant differences (*p* > 0.05).

**Conclusions:**

We observed significant correlations between different imaging findings suggestive of vasculitis on ultrasound or MRI and the final adjudicated diagnosis. While ultrasound and MRI were considered suitable imaging methods for detecting and discriminating typical vascular changes, ^18^F-FDG PET/CT requires careful timing and patient selection given its moderate diagnostic accuracy.

## Highlights

• MRI and ultrasound provided significant correlations between findings suggestive of vasculitis and the final diagnosis.
• Careful selection of available imaging techniques is warranted considering the time course, location, and clinical history.
• Considering its moderate diagnostic power to distinguish tracer uptake, a holistic view of PET/CT findings is essential.

## Nomenclature

^18^F-FDG PET/CT: ^18^F-fluorodeoxyglucose positron emission tomography/ computed tomography.
AAV: ANCA-associated small-vessel vasculitis.
ANA: Antinuclear antibodies.
ANCA: Antineutrophil cytoplasmatic antibodies.
AUC: Area under the curve.
BD: Behçet disease.
CRP: C-reactive protein.
GCA: Giant cell arteritis.
GPA: Granulomatosis with polyangiitis.
IQR: Interquartile range.
MPA: Microscopic polyangiitis.
PAN: Periarteritis nodosa.
PET/CT: Positron emission tomography/computed tomography.
ROC: Receiver operating characteristic.
SD: Standard deviation.
SUV: Standardized uptake value.
MC: Mixed cryoglobulinemia.

## 1 Introduction

Vasculitis represents a collective term for miscellaneous complex and heterogeneous vascular diseases characterized by vessel wall inflammation, finally resulting in the development of occlusive and aneurysmal vascular lesions accompanied by significant morbidity [1]. It is a rare disorder of largely unknown etiology with an annual incidence of up to 60/million in those aged 65–74 years across different ethnicities [2], [3].

According to the revised nomenclature adopted at the Chapel Hill Consensus Conference [4], vasculitis is divided into primary (e.g., autoimmune) or secondary forms as a result of connective tissue diseases, drugs, infections, or malignancies. Clinical classification is based on vessel size allowing for the differentiation between vasculitis of large vessels (e.g., giant cell arteritis (GCA)), medium vessels (e.g., panarteritis nodosa (PAN)), and small vessels with immune complex deposits (e.g., mixed cryoglobulinemia (MC)) or antineutrophil cytoplasmatic antibodies (ANCA) (e.g., granulomatosis with polyangiitis (GPA) or microscopic polyangiitis (MPA)) [5], [6], [7]. However, some types may affect vessels of variable sizes, such as Behçet disease (BD) [8]. Whereas large vessel vasculitis typically shows an inflammatory response starting at the adventitia, small vessel inflammation spreads from the endothelial layer outward to the media and adventitia [9]. Early activation of the coagulation system and fibrinolysis in small vessels ultimately leads to thrombotic and necrotizing complications contributing to the progression of cardiovascular diseases [6], [10], [11].

GCA is the most common form of systemic vasculitis, usually with a self-limiting clinical course [12]. However, a few patients may take a chronic course with symptoms lasting for years, frequently experiencing relapses that require indefinite treatment with glucocorticoids [12]. Survival studies of patients with GCA have found mortality rates that were comparable with those of the general population, but at an increased risk for potentially life-threatening ischemic events [13]. Therefore, identifying early morphological changes remains one of the most important goals not only for making the right diagnosis but also for assessing therapeutic response and detecting disease relapse. Since treatment of systemic vasculitis varies from other types of vasculopathy, a high diagnostic accuracy seems to be crucial [14].

This study sought to assess the diagnostic value of the imaging modalities ultrasound, magnetic resonance imaging (MRI), and ^18^F-fluorodeoxyglucose positron emission tomography/computed tomography (^18^F-FDG PET/CT) in distinguishing systemic vasculitis from other internal and immunological diseases.

## 2 Materials and methods

The present study was approved by the local ethical review board. The requirement to obtain written informed consent was waived.

### 2.1 Study population

**Fig. 1:**
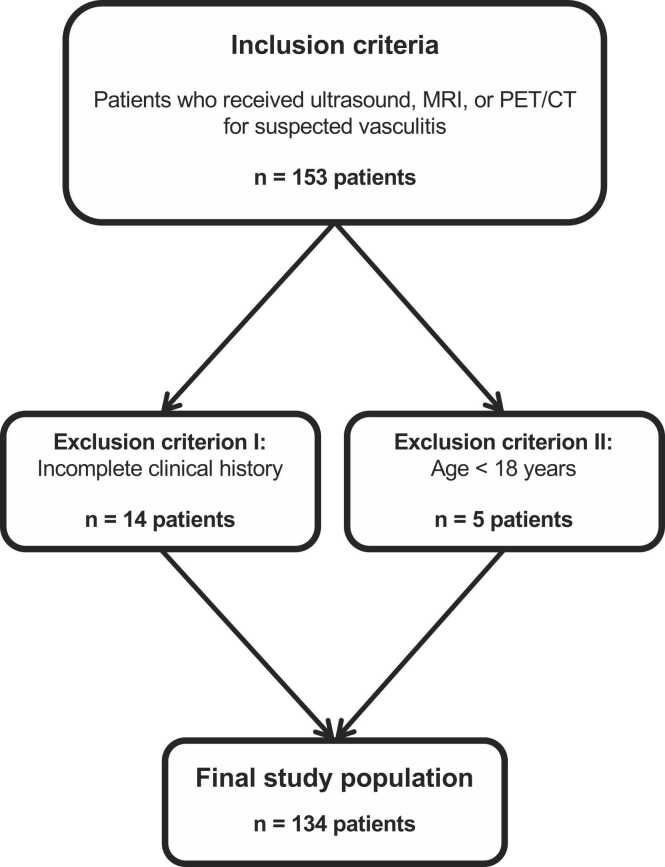
Illustration of patient inclusion.

In this retrospective work, a total of 153 patients who received an imaging procedure (ultrasound, MRI, or ^18^F-FDG PET/CT) for suspected vasculitis in the period from 01/2010–01/2019 were initially considered for study inclusion. Given its clinical heterogeneity, features suggestive of vasculitis consisted of systemic symptoms (e.g., fatigue, fever, weight loss, or arthralgia), findings in physical examination (e.g., motor weakness of the extremities due to neuropathic disorder, blood pressure discrepancies, or palpable purpura), and laboratory testing (antinuclear antibody test, serum complement levels, or antineutrophil cytoplasmatic autoantibodies). The study was performed at the University Hospital Frankfurt (Frankfurt am Main, Hesse, Germany). 19 patients were excluded due to incomplete clinical history (n = 14) or patient age < 18 years (n = 5). Finally, datasets from 134 patients were analyzed (Fig. 1).

### 2.2 Patient data

Patient data were collected from electronic medical reports and analyzed using the Picture Archiving and Communication System (PACS Centricity 4.2; General Electric Healthcare, Chicago, USA). All available data were evaluated, including visit entries, doctor's letters, entries from consultations at outpatient clinics, results of histopathological examinations, and admission forms. Extracted data also included sex, age, previous illnesses of the patient, presence of already known vasculitis, possible previous therapy for vasculitis, symptoms, laboratory values, additional examinations, therapy, and follow-up data. The final adjudicated diagnosis of vasculitis was based on clinical and histopathological findings, which were available in all cases.

Furthermore, follow-up of patients with a final diagnosis of vasculitis was registered. Defined follow-up periods were examined, which could be divided into the following three categories: up to 6 months, 6–12 months, and 12–24 months after the first presentation at the University Hospital Frankfurt.

The main study parameter was the confirmation of the diagnosis employing the three different imaging modalities, with the clinical and histopathological diagnosis as the gold standard. Secondary parameters were the morphological characteristics of vessels affected by vasculitis as visualized with ultrasound, MRI, and PET/CT.

All imaging data were retrospectively analyzed by a board-certified radiologist with 16 years of experience (T.G.-R.).

### 2.3 Ultrasound

A total of 98 patients underwent sonography using an "xVision" device (MyLab70xVision; Esaote Biomedica, Cologne, Germany). Depending on the region and vessel to be examined, the appropriate transducer was selected by the examiner (K.E., board-certified radiologist with 14 years of experience in ultrasound).

The following data were collected and evaluated for each patient undergoing ultrasound: date, region, total number of findings, structure of the vessel wall, and affected vessels. Regarding vessel wall characteristics, the examiners have also evaluated hypoechoic vessel wall thickening around the lumen, which was termed “halo sign” and suggested as pathognomonic for temporal arteritis. Vascular edema was diagnosed as soon as hypoechoic black fluid signal alterations were visible.

### 2.4 Magnetic resonance imaging

The indication to perform an MRI examination in case of suspected vasculitis was set by the treating physician based on clinical and laboratory findings. The MR examination was performed in the supine position using a 3-Tesla MRI (Prisma fit; Siemens Healthineers, Forchheim, Germany). The contrast agent was administered via peripheral venous access (Gadovist™ [EU]). Depending on the region to be examined, a dedicated body coil was placed (e.g., a 16-channel body coil).

All MRI examinations contained basic fat-suppressed T1-weighted sequences, T2-weighted turbo spin echo sequences, as well as routinely included "dark-blood sequences" ("db sequence", navigated 3D turbo spin echo) for a better assessment of mural inflammation. All images were acquired in axial orientation except for T2-weighted sequences, which were also performed in the coronal plane.

The following data were evaluated: date, region, affected vessels, edema or thickening of the vessel wall, diameter of the vessel wall and lumen, fluid deposition around the vessel, and the presence of aneurysms. The vessel wall was measured at the thickest point in the axial plane, whereas the wall of the aortic arch was assessed in the coronal plane.

### 2.5 Positron emission tomography/computed tomography

Before performing ^18^F-FDG PET/CT, each enrolled patient was carefully instructed by the attending physician. The interval between FDG infusion and image acquisition was at least 60 minutes. All scans were performed in the supine position with arms down (Biograph 6; Siemens Healthineers), as previously described [15]. The scan area included the whole body from the base of the skull to mid-thigh.

The following data were analyzed: date, affected vessels including vessel thickening and diameter, tracer uptake of the vessel, and maximal standardized uptake values (SUV_max_). In this context, FDG uptake was either assessed by using a qualitative visual grading score (range, 0–3) or a semiquantitative approach by measuring standardized uptake values. For semiquantitative assessment, regions of interest were manually drawn around the affected vessel wall in consecutive axial slices.

### 2.6 Statistical analysis

Statistical analysis was performed with dedicated software (MedCalc, Version 19; Ostend, Belgium). All data sets were checked for normal distribution using the Kolmogorov-Smirnov test. Variables were expressed as means ± standard deviation (SD), count (percentage), or median (interquartile range, IQR), where appropriate. A *p-*value of less than 0.05 was considered statistically significant.

Comparisons between continuous variables were conducted using one-way ANOVA, chi-square statistic tests, or two-tailed Student’s t-test, where appropriate. Receiver operating characteristic (ROC) curves were plotted and areas under the curves (AUCs) were calculated together with their 95% confidence intervals (95% CI). Correlations were assessed using Spearman’s correlation coefficient. A descriptive analysis of follow-up imaging was performed, evaluating how many examinations the patients received in a defined observational period.

## 3 Results

A total of 134 patients with suspected vasculitis and corresponding imaging were included in this retrospective analysis, consisting of 81 women (60%) and 53 men (40%) with an average age of 65 ± 17 years (range, 19–90 years). Among these, the vasculitis group included 29 men and 41 women with a mean age of 68 ± 14 years (range, 19–90 years). The comparative non-vasculitis group was composed of 24 men and 40 women with a mean age of 61 ± 19 years (range, 22–85 years).

The division of the patients into two groups was based on the final adjudicated diagnosis as derived from clinical and histopathological findings. While 70 patients (52%) were diagnosed with a type of vasculitis (mostly GCA in 47% of all cases), the remaining 64 patients (48%) served as the comparative group showing miscellaneous conditions, including 47 patients with internal and immunological diseases, such as coronary heart disease, pneumonia, inflammatory bowel disease, polyserositis, or unspecified forms of immunodeficiency.

Of the 134 patients, the largest group of patients (n = 52, 39%) suffered from cardiovascular disease. Rheumatic diseases were diagnosed in 24 patients (18%), whereas some form of vasculitis was already known in 19 patients (14%). Malignant diseases were present in 21 patients (16%).

Baseline characteristics are summarized in Table 1.

**Table 1:** Baseline characteristics of the study cohort.

Variables	**Vasculitis** (n = 70; 52%)	**Non-vasculitis** (n = 64; 48%)	***p*-value**
**Demographics**			
Age (years) ± SD, range	68 ± 14 (19–90)	61 ± 19 (22–85)	0.0159
Males (n)	29 (41%)	24 (38%)	
Females (n)	41 (59%)	40 (62%)	
**Pre-existing disease**			
Cardiovascular disease (n)	34 (49%)	18 (28%)	
Known vasculitis (n)	19 (27%)	–	
Cancer (n)	12 (17%)	9 (14%)	
Metabolic disease (n)	10 (14%)	7 (11%)	
Chronic infection (n)	9 (13%)	5 (8%)	
Rheumatic disease (n)	7 (10%)	17 (27%)	
Neurologic disorder (n)	7 (10%)	3 (5%)	
**Symptoms**			
Aggravated vasculitis (n)	18 (26%)	–	
Headache (n)	17 (24%)	–	
Visual disturbance (n)	17 (24%)	7 (11%)	
Joint pain (n)	15 (21%)	25 (39%)	
Fever (n)	10 (14%)	10 (16%)	
Deterioration of general condition (n)	9 (13%)	7 (11%)	
Muscle pain (n)	8 (11%)	15 (23%)	
Pain in the chewing muscles (n)	7 (10%)	–	
Weight loss (n)	–	10 (16%)	
**Imaging**			
Ultrasound (n)	51 (73%)	47 (73%)	
Magnetic resonance imaging (n)	69 (99%)	64 (100%)	
^18^F-FDG PET-CT (n)	18 (26%)	9 (14%)	
**Final diagnosis of vasculitis**			
Giant cell arteritis (n)	33 (47%)	–	
Unspecified vasculitis (n)	15 (21%)	–	
Aortitis (n)	11 (16%)	–	
Takayasu arteritis (n)	6 (9%)	–	
Granulomatosis with polyangiitis (n)	2 (3%)	–	
Panarteritis nodosa (n)	1 (1%)	–	
Morbus Behçet (n)	1 (1%)	–	
Small-vessel vasculitis (n)	1 (1%)	–	

### 3.1 Symptomatology

Comprising all patients, the most common symptom reported by 40 patients (30%) was joint pain. 24 patients (18%) complained of visual disturbances and 23 patients (17%) of muscle pain. Increased symptomatology brought 18 patients (13%) with known vasculitis to the hospital. 18 patients (13%) reported neurocranial and visceral pain. Another 18 patients (13%) were grouped under the heading of other symptoms, including unspecific abdominal pain or atypical chest pain. Deterioration of patients' general condition was reported by 16 patients (12%).

### 3.2 Laboratory

Overall white blood cell count was 10.28 × 10^9^/L (range, 4.24–23.36 ×10^9^/L) and differed nonsignificantly between patients with and without vasculitis (10.18 ×10^9^/L vs. 10.38 ×10^9^/L, *p* > 0.05). The AUC was 0.509 for the discrimination of patients with vasculitis from the comparative group (95% CI, 0.41–0.61; *p* = 0.86).

Values for the blood sedimentation rate showed an AUC of 0.595 (95% CI, 0.49–0.70; *p* = 0.08), without differences between the two groups (*p* > 0.05).

Overall C-reactive protein (CRP) was 4.9 mg/l (range, 0.02–28.21 mg/l), nonsignificantly differing between the vasculitis and non-vasculitis group (5.27 vs. 4.49 mg/l; *p* > 0.05). ROC analysis showed an AUC of 0.553 (95% CI, 0.45–0.65; *p* = 0.29).

A determination of antineutrophil cytoplasmatic antibodies (cANCA) was performed in 75 of 134 patients (56%). A total of 6 patients (8%) was tested positive, with 2 patients in the vasculitis and 4 patients in the non-vasculitis group. Antinuclear antibodies (ANA) were determined in 88 of 134 patients (66%). Positive testing was observed in 55 patients (63%), of whom 25 patients belonged to the vasculitis and 30 patients to the non-vasculitis group.

Values of interleukin-6 (IL-6) were 110 ng/l on average, determined in 15 of 134 patients (11%). Average values of 146 ng/l and 40 ng/l were measured in the vasculitis and non-vasculitis group, respectively (*p* < 0.05).

### 3.3 Ultrasound

Ultrasound was performed in 98 of 134 patients (73%), with 51 patients (52%) in the vasculitis and 47 patients (48%) in the non-vasculitis group. The majority of 70 patients (71%) received a duplex ultrasound of the extracranial cerebral arteries. Pelvic and leg arteries were examined in 13 patients (13%), abdominal arteries in 11 patients (11%), arm arteries in 3 patients (3%), and supra-aortic arteries in 1 patient (1%). Ultrasound revealed a variety of different vascular findings, mostly hyperechoic plaques in 26 patients (27%) and "halo sign" in a total of 16 patients (16%). 41 patients (42%) did not show any vascular changes.

Vascular findings of "halo sign", "vascular occlusion", "thrombosis with vascular occlusion", and "atypical vascular thickening" were found in 31 of 98 patients (32%) and can be regarded as indicative of vasculitis together with typical clinical signs and symptoms. This resulted in a Chi² of 5.45 with a *p*-value of 0.02. Thus, a significant relationship between vascular findings on ultrasound and the final clinical diagnosis could be demonstrated.

**Fig. 2:**
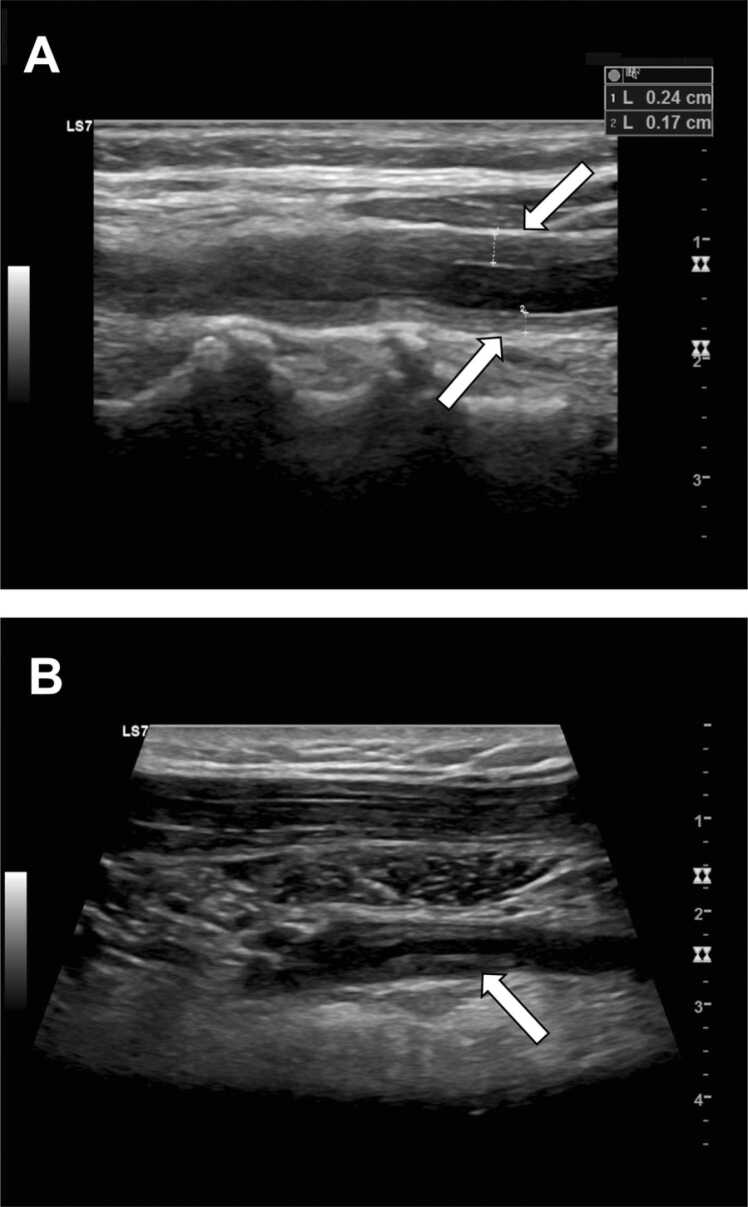
Illustration of grayish, hypoechoic vessel wall thickening with a width of 0.24 cm at the top and 0.17 cm at the bottom in the longitudinal section of the left common carotid artery *(white arrows) **(A)***. Vascular edema is visible as a hypoechoic, black fluid signal *(white arrow)* within the plaques ***(B)***.

Fig. 2A illustrates the typical homogeneously grayish, hypoechoic vessel wall thickening with a width of max. 0.24 cm in the longitudinal section of the left common carotid artery *(white arrows)*. Vessel wall findings of "edema" and "hyperechoic plaques" in 43 patients (44%) could also be interpreted as suggestive of vasculitis, resulting in a Chi² of 4.37 with a *p*-value of 0.04. Vascular edema was diagnosed as soon as hypoechoic, black fluid signals *(white arrow)* were visible in the plaques, as illustrated in Fig. 2B.

In the vasculitis group, 15 patients (29%) had hyperechoic plaques and 9 patients (18%) hypoechoic plaques. 14 patients (28%) showed no affected vessel in the examined area. In 15 patients (29%), the common carotid artery was affected, followed by the superficial temporal artery (n = 8, 16%) and internal carotid artery (n = 4, 8%). Finally, the diagnosis of a form of vasculitis could be established in a total of 18 patients by ultrasound.

### 3.4 Magnetic resonance imaging

A total of 133 patients (99%) underwent MRI, of whom 69 patients (52%) belonged to the vasculitis and 64 (48%) to the non-vasculitis group. The majority of 76 patients (57%) showed no vascular affection suggestive of vasculitis.

Vessel wall alterations suggestive of vasculitis were visible in a total of 57 patients (43%), 48 (84%) of whom belonged to the vasculitis group, showing a vessel wall thickness of 5.05 mm on average (range, 2–12 mm). A total of 31 patients (54%) presented an affection of the descending thoracic aorta, whereas 12 patients (21%) additionally showed an affected abdominal aorta. In the non-vasculitis group, 9 patients (16%) showed vascular alterations that were initially interpreted as vasculitis but finally discarded in consideration of the clinical and histopathological results. Wall thickness was 5.14 mm on average, ranging from 3 to 8 mm (*p* < 0.0001 vs. vasculitis group).

**Fig. 3:**
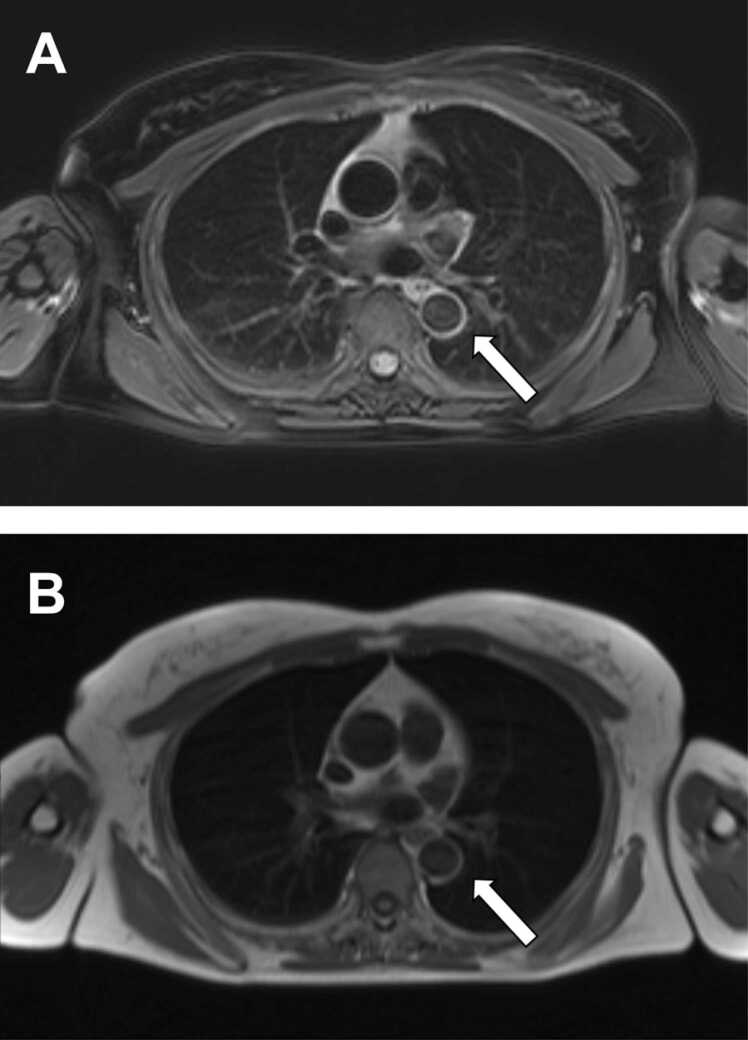
On MRI, vessel wall edema was observed in a total of 28 patients (21%). Correspondingly, axial non-contrast T2-weighted MR images ***(A)*** and T1-weighted fat-suppressed MR images ***(B)*** showed a hyperintense circular cuff around the thoracic aorta *(white arrows)*.

Vessel diameter differed significantly between patients with finally diagnosed vasculitis (mean of 26.4 mm, range 6–40 mm) and non-vasculitis (mean of 30.1 mm, range 20–44 mm; *p* < 0.0001). Vessel wall thickening and diameter demonstrated a significant correlation of 0.61 (*p* < 0.0001). Wall edema was observed in a total of 28 patients (49%), 25 and 3 of whom belonged to the vasculitis and non-vasculitis group, respectively (Fig. 3).

### 3.5 Positron emission tomography/computed tomography

^18^F-FDG PET/CT was performed in 27 of 134 patients (20%). 18 examinations (67%) were carried out in the vasculitis group and 9 (33%) in the non-vasculitis group.

In the majority of 17 patients (63%) no affected vessel could be identified. Tracer uptake in the vessel wall could be detected in 10 patients (37%), 8 of whom belonged to the vasculitis group. Vasculitis of the descending and abdominal aorta was detected in 3 and 2 cases, respectively.

**Fig. 4:**
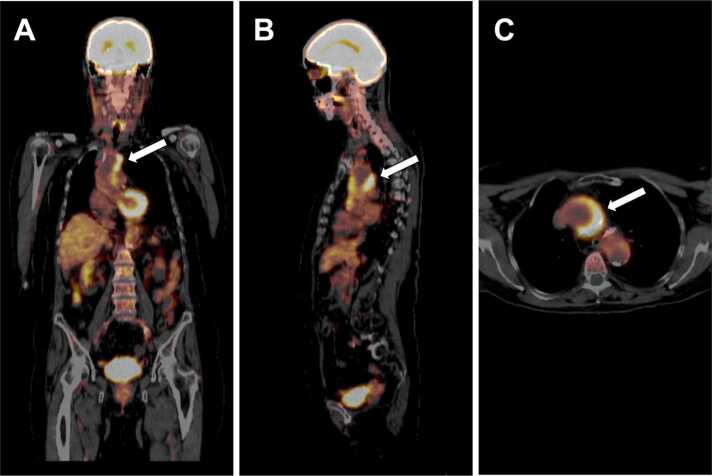
Illustration of a 72-year-old patient with newly diagnosed aortitis who initially presented to the emergency department with atypical chest pain, fatigue, and fever caused by systemic inflammation. Regions with the strongest ^18^F-fluorodeoxyglucose uptake are marked by a white arrow.

**Fig. 5:**
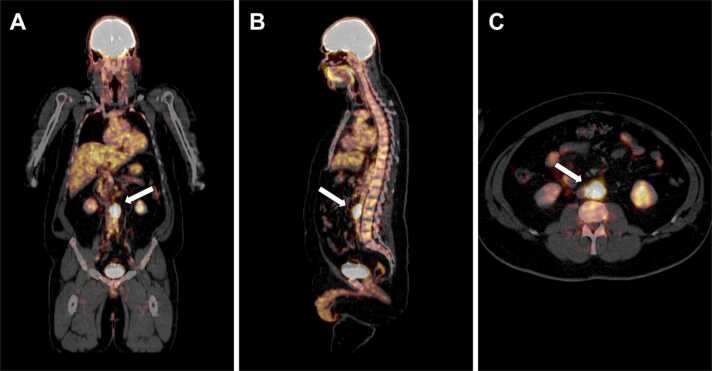
A 48-year-old patient who was admitted to the emergency department with persistent flank and back pain in the lumbar spine area. In an initial CT scan of the lumbar spine, an unknown tissue formation was accidentally discovered which was located around the infrarenal abdominal aorta. A clear tracer uptake *(white arrows)* has been observed in a subsequent PET/CT examination, finally establishing the diagnosis of large-vessel vasculitis.

Mean SUV_max_ values were 5.2 (range, 2.8–10.2) in the vasculitis group and 9.8 (range, 3.3–16.2) in the non-vasculitis group, respectively (*p* = 0.49). Correlation of SUV_max_ values with vessel wall thickening and vessel diameter revealed coefficients of r = 0.46 (*p* = 0.36) and r = 0.14 (*p* = 0.80), respectively. Fig. 4 shows a case example of a 72-year-old patient with newly diagnosed aortitis who initially presented with atypical chest pain, fatigue, and fever caused by systemic inflammation. Another case is illustrated in Fig. 5 showing a 48-year-old patient who was admitted to the emergency department with persistent pain radiating to the lumbar spine. A clear tracer uptake *(white arrows)* was discovered around the infrarenal abdominal aorta in a subsequent PET/CT examination which finally led to the diagnosis of large-vessel vasculitis.

### 3.6 Additional examinations and imaging follow-up

Additional examinations other than ultrasound, MRI, and ^18^F-FDG PET/CT were performed in 59 of 134 patients (44%), including angiographies or consultations in departments of ophthalmology, rheumatology, and neurology. This resulted in 7 additional cases with a final diagnosis of vasculitis, which otherwise would have been missed underestimating the final adjudicated diagnosis by approximately 10%.

The time between final diagnosis and imaging follow-up of the individual patient was variable, depending on patient compliance and place of follow-up. In this context, follow-up imaging was not available for the majority of patients (n = 87, 65%), including 32 patients (37%) from the vasculitis group.

A total of 86 patients (64%) continued to be cared for in the rheumatologic outpatient clinic at the University Hospital Frankfurt. 19 patients (14%) received another follow-up check in an external hospital or medical office.

## 4 Discussion

Systemic vasculitis comprises a heterogeneous group of conditions characterized by inflammation of blood vessels [1]. It typically has manifold manifestations, such as joint paint, which was the most common symptom in our study. We found significant correlations between different imaging findings suggestive of vasculitis and the final adjudicated clinical and histopathological diagnosis. In this context, on MRI, vessel wall thickening, edema, and diameter differed significantly between the vasculitis and non-vasculitis group (*p* < 0.05). Ultrasound revealed different findings that may serve as red flags in identifying patients with vasculitis, such as vascular occlusion, vascular thrombus formation, or the so-called “halo sign”. Interestingly, comparing SUV_max_ values from PET/CT examinations with vessel wall thickening and vessel diameter did not result in significant differences (*p* ≥ 0.36).

Along with technical developments over the past decades, many forms of vasculitis can be visualized either directly by MRI, PET/CT, and ultrasound, or indirectly by identifying sequelae of inflammatory processes [16]. The choice of the most appropriate imaging modality significantly depends on the location and size of the affected vessel. While the assessment of very small vessels commonly remains frequently non-diagnostic due to poor resolution, indirect signs of the adjacent inflammatory response may be visualized as being suggestive of possible vascular affection [6]. Consequently, extracranial arteries and main aortic branches should preferably be examined using MRI or CT, whereas high-resolution ultrasound can be recommended for the examination of smaller arteries, such as temporal arterial branches.

Serological determination of ANCAs is recommended as the preferred first screening method for ANCA-associated small-vessel vasculitis (AAV) [17]. Additionally to elevations in patients with AAV, ANCAs are also pathologically altered in miscellaneous conditions, such as gastrointestinal disorders, rheumatoid arthritis, systemic lupus erythematosus, or infections [18]. In our study, a determination of cANCAs was performed in 75 of 134 patients, with in total 6 positive cases (4 positive cases in the non-vasculitis group). In light of concomitant comorbidities and patient history, the elevations in the non-vasculitis group can be interpreted as false positive results. Follow-up analysis revealed underlying primary sclerosing cholangitis (n = 1), Crohn’s disease (n = 1), and ulcerative colitis (n = 2).

While characteristically associated with autoimmune connective tissue diseases, ANA elevations may also be observed in several non-rheumatological conditions, like malignancies, infections, skin diseases, or other autoimmune diseases [19], [20]. Conclusively, ANA testing is an important tool in the diagnosis of rheumatological diseases but has limited value in the absence of clinical correlation. Hence, awareness regarding the judicious use of the same is imperative in clinical practice [21]. To avoid false positive clinical interpretation of test results in the context of significant interfering comorbidities and diseases, the assessment of antibody testing should be made by knowledge of patient history, current active diseases, medication, and other circumstances that may affect the test result. In our study, ANAs were determined in 88 of 134 patients, with 25 and 30 positive tests in the vasculitis and non-vasculitis groups, respectively.

Inflammation of blood vessels with concomitant vessel thickening, thrombus formation, and luminal occlusive remodeling is accompanied by ischemia, pain, and finally malfunction of the affected organ. IL-6 represents a pleiotropic pro-inflammatory cytokine that induces inflammatory response and is decisively involved in immune regulation, metabolic processes, and differentiation of cells [22]. High plasma concentrations of IL-6 are regarded to be responsible for the symptoms associated with inflammatory vessel damage in affected patients [23], [24]. Corresponding with previous studies [22], [25], [26], average IL-6 values of 146 ng/l and 40 ng/l were measured in the vasculitis and non-vasculitis groups, respectively.

Vasculitis in its various forms can potentially affect arteries and veins of all sizes in different parts of the body. While ultrasound and MRI are considered suitable imaging methods for detecting and discriminating vascular changes in comparison to non-affected patients, PET/CT examinations were not able to detect affected vessels in some cases. In general, ^18^F-FDG-PET/CT has only moderate diagnostic accuracy to discriminate between patients with active disease and those in clinical remission, showing a pooled sensitivity and specificity of 77% (95% CI, 57–90%) and 71% (95% CI, 47–87%), respectively [27]. In 9 patients with tracer accumulation in the vessel wall, SUV_max_ values differed nonsignificantly between the vasculitis and non-vasculitis groups, respectively (*p* = 0.49). Additionally, correlations of SUV_max_ with vessel wall thickness and diameter remained nonsignificant (*p* ≥ 0.36). As uptake of ^18^F-FDG commonly decreases upon clinical remission in patients treated for vasculitis disease, ^18^F-FDG PET/CT is not recommended for rule in or rule out of the disease [27]. Interestingly, two patients in the non-vasculitis group with prominent focal atherosclerotic plaque burden showed pronounced ^18^F-FDG vessel uptake, which highlights the limitations of this diagnostic modality. As soft atherosclerotic lesions might influence tracer uptake, discriminating between atherosclerotic and vasculitic lesions may sometimes be challenging in daily clinical routine [28], [29]. Therefore, great care must be taken regarding the patient’s history and timing of examinations, especially in consideration of the frequently limited accessibility of this diagnostic technology.

In this context, data on ^18^F-FDG PET/MRI as a new hybrid imaging modality in the detection and follow-up of patients with vasculitis are sparse. However, PET/MRI seems to reach comparable visual and quantitative results compared to ^18^F-FDG PET/CT, showing significant correlations with clinical findings [30], [31], [32], [33], [34], [35]. Given the need to reduce radiation exposure in young patients and those undergoing lifelong serial follow-up examinations, PET/MRI offers a viable alternative to PET/CT [34], [35]. Another important point arises from the need to identify subclinical smoldering disease potentially resulting in a complicated course in case of a delayed diagnosis. In this context, the high soft tissue contrast of MRI may improve the diagnostic accuracy in detecting less marked imaging findings suggestive of vasculitis [36]. However, the reliability and accuracy of the different imaging modalities in detecting vasculitis and assessing disease activity or therapeutic response should be subject to future prospective trials. With the advent of more sophisticated imaging modalities and improved technology, careful consideration is required for choosing the most appropriate imaging method.

Several limitations have to be addressed when interpreting our study results. First, the present study was retrospective with a limited number of patients requiring prospective validation in larger trials. Second, the study was performed with vendor-specific setups not comprising devices from other manufacturers. Especially the investigation of new-generation CT and MRI engines with the ability to visualize even small-sized arteries at high image quality seems to remain one of the most important goals for improving the early detection of patients with vasculitis. Third, laboratory values were determined at the presentation of patients without considering time-dependent changes. Finally, some PET/CT examinations were performed under immunosuppressive therapy which potentially resulted in false-negative results.

In conclusion, our study revealed significant correlations between different imaging findings suggestive of vasculitis on ultrasound and MRI and the final adjudicated clinical and histopathological diagnosis. While ultrasound and MRI were considered suitable imaging methods for detecting and discriminating vascular changes in comparison to non-affected patients, PET/CT examinations were not able to detect affected vessels in some cases due to moderate diagnostic accuracy in the discrimination of patients with active disease.

## Funding statement

No funding has been received for this project.

## Ethical statement

The present work has been carried out in accordance with The Code of Ethics of the World Medical Association (Declaration of Helsinki) and is in line with the Recommendations for the Conduct, Reporting, Editing and Publication of Scholarly Work in Medical Journals. Moreover, the work aims for the inclusion of representative human populations (sex, age, and ethnicity) as per those recommendations. The terms sex and gender are used correctly.

The institutional ethical review board approved this retrospective study. The need for written informed consent was waived. The privacy rights of human subjects have been always observed.

## CRediT authorship contribution statement

**VK:** Writing – original draft, Supervision, Project administration, Software. **JA, KE, MH, and LDG:** Data curation, Investigation. **TDA, JA, and SSM:** Visualization, Resources. **KE, TDA, MA, AT, CB, IY, SB, and SM:** Formal analysis, Methodology, Software, Validation. **SM, AT, IY, MH, JA, WD and DG:** Project administration, Validation. **TJV, GJ and TGR:** Writing – review & editing, Methodology, and Conceptualization. All authors have reviewed and agreed with the content of the article.

## Conflicts of interest

I.Y. received a speaking fee from Siemens Healthineers. C.B. received speaking fees from Siemens Healthineers. The other authors have no potential conflict of interest to disclose.
